# Squamous cell carcinoma of the uterine cervix metastasising to the thyroid gland: a case report

**DOI:** 10.1186/s40792-021-01341-1

**Published:** 2021-12-15

**Authors:** Sanjeevan Ravindrakumar, Nanduni Thalahitiyage, Nagenthiram Harivallavan, Umesh Jayarajah, Vitharanage Srimantha Dewsiri Rodrigo

**Affiliations:** Department of Surgery, District General Hospital Chilaw, Putlam, Chilaw, Sri Lanka

**Keywords:** Carcinoma uterine cervix, Cervical cancer, Thyroid metastasis, Squamous cell carcinoma, Case report

## Abstract

**Background:**

Carcinoma of the uterine cervix (cervical cancer) metastasising to the thyroid gland is a rare phenomenon and only a few cases have been reported. We discuss a patient with cervical cancer presenting with thyroid and cervical lymph node metastasis, exploring the diagnostic difficulty, evaluation and treatment options.

**Case presentation:**

A previously well 56-year-old female presented with multiple neck lumps for 4 months duration. Examination of the neck revealed multiple firm/hard left cervical lymph nodes with a hard thyroid nodule. There were no abdominal masses however, vaginal examination revealed a hard, unhealthy cervix. Contrast enhanced computed tomography revealed a growth in the uterine cervix with lymph node enlargement in the abdomen, chest and neck along with multiple thyroid nodules. Biopsy of the cervix and cervical lymph node and fine needle aspiration cytology of the thyroid nodules were performed, all revealing a squamous cell carcinoma. Pan-endoscopy performed to rule out any concurrent cancer of the upper aerodigestive tract was negative. The patient was started on palliative therapy, but succumbed to the disease after 6 months.

**Discussion and conclusion:**

Patients who present with a thyroid nodule along with multiple cervical lymph nodes should be thoroughly assessed for possible metastatic cancers. Metastasis to the thyroid gland indicates a poor prognosis in the background of carcinoma or the uterine cervix. More awareness among the public and primary care providers is necessary regarding freely available screening programmes for early detection of cervical cancer.

## Background

Metastatic thyroid tumours are extremely rare [1]. Breast, lung and renal cell cancers are known primaries that can give rise to thyroid metastasis [1]. Lymphatic spread of carcinoma of the uterine cervix to cervical lymph nodes and thyroid gland is an unusual mode of presentation [2]. In this case report, we describe a female who presented with multiple neck lumps. Further evaluation revealed that these lumps were metastatic deposits of squamous cell carcinoma of the uterine cervix to the cervical lymph nodes and the thyroid gland.

## Case presentation

A 56-year-old woman, a mother of 3 children, presented to the surgical department with a history of multiple neck lumps of 4 months duration. She also had generalised vague abdominal pain, loss of appetite and lower back pain. She had no significant medical, family or psychosocial history. Clinical examination revealed multiple, bilateral enlarged cervical lymph nodes which were firm to hard in consistency. Thyroid examination revealed a 2 × 2 cm firm lump on the lower pole of the left thyroid lobe. Examination of other lymph node groups revealed enlarged right inguinal lymph nodes. Abdominal examination and rectal examination were normal. Vaginal examination revealed a hard, unhealthy uterine cervix. Breast and axillary examination were unremarkable. She had spinal tenderness, but the neurological examination of the lower limbs was normal.

**Fig. 1:**
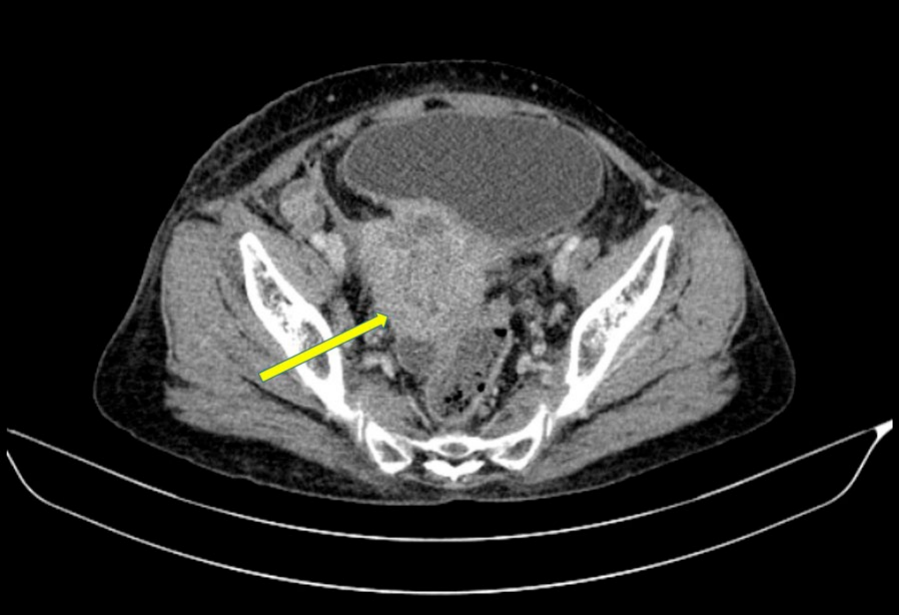
Axial view of the contrast enhanced computed tomography of the pelvis showing an irregular heterogenous mass arising from the uterine cervix (yellow arrow)

**Fig. 2:**
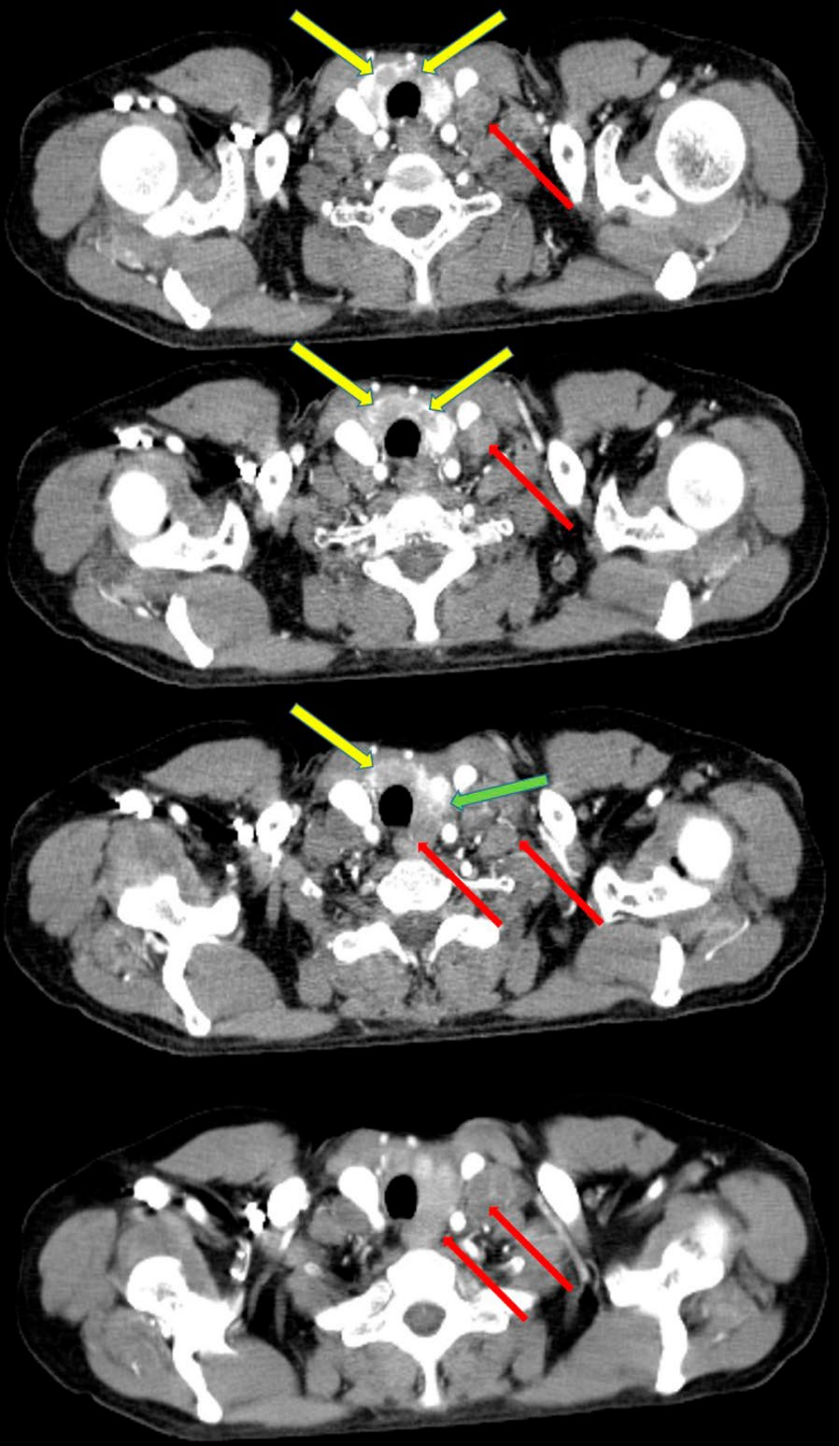
Consecutive axial views of the contrast enhanced computed tomography of the neck showing multiple low-density thyroid nodules (yellow arrows), a mixed density mass lesion in the lower pole of the left thyroid lobe (green arrow) and multiple deep cervical lymph nodes (lower jugular and paratracheal) (red arrows)

Her basic blood investigations, liver profile and renal functions were within the normal limits. Ultrasound scan of the abdomen revealed no abnormalities. Ultrasound scan of the neck revealed multiple nodules in the thyroid, with increased vascularity, and multiple enlarged cervical lymph nodes with obliterated fatty hila suggestive of malignant deposits. Contrast enhanced computed tomography of the neck, chest, abdomen and pelvis showed a mass in the uterine cervix (Fig. 1) with multiple enlarged lymph nodes in the inguinal, iliac, para-aortic, anterior mediastinal and bilateral deep cervical groups (Fig. 2). There were multiple low-density nodules in the thyroid gland (Fig. 2). A mixed density mass lesion was also noted in the lower pole of the left thyroid lobe (Fig. 2). Furthermore, an anterior wedge fracture of the L2 vertebra was seen, probably secondary to bone metastases.

**Fig. 3:**
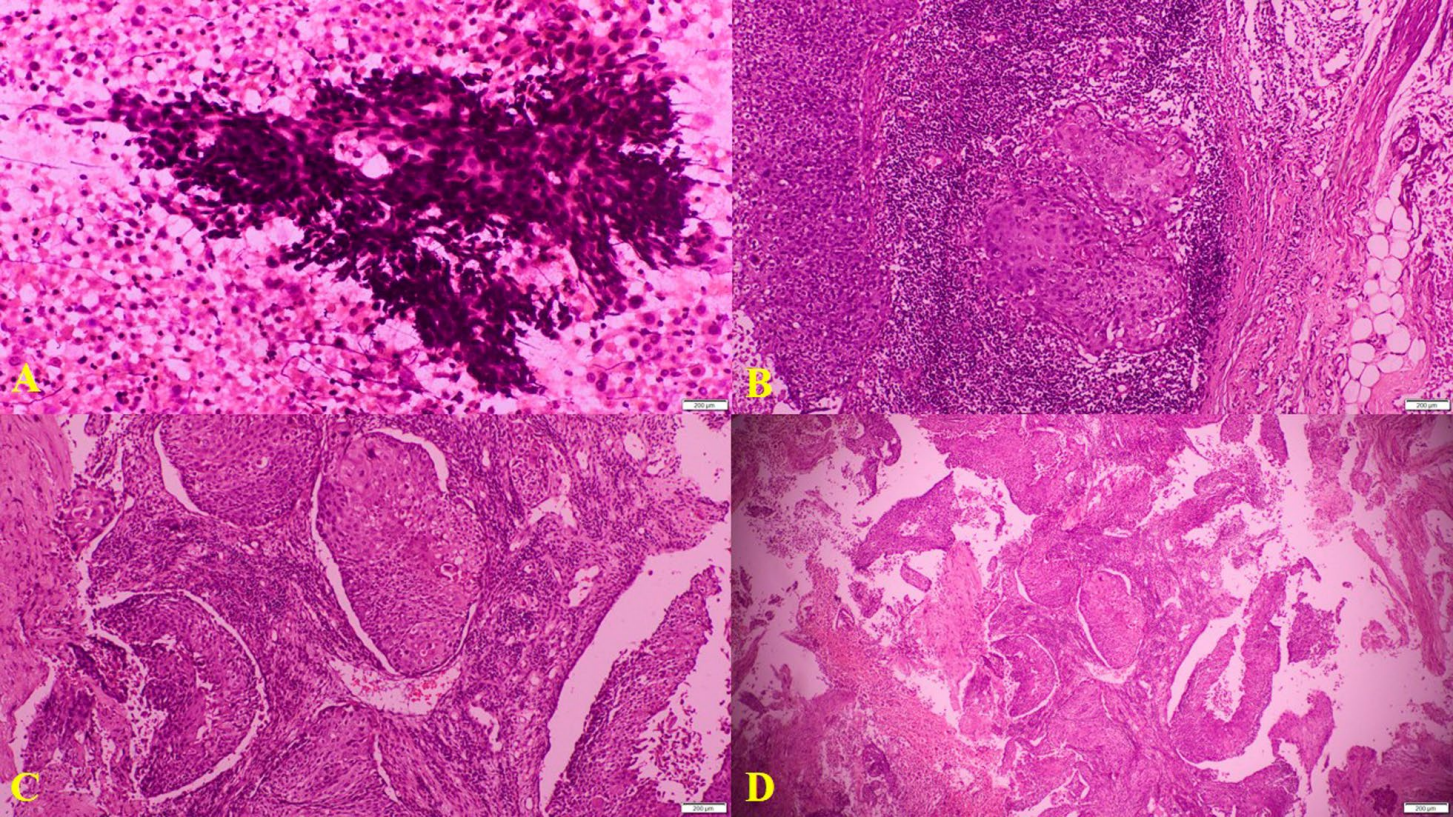
H&E stains. **A** Ultrasound-guided fine needle aspiration of the thyroid nodules showing squamous cell carcinoma. **B** Cervical lymph node biopsy showing metastatic squamous cell carcinoma. **C** Biopsy of the uterine cervix showing a moderately differentiated squamous cell carcinoma. **D** Endometrial curettage showing a moderately differentiated squamous cell carcinoma

Biopsies from the uterine cervix and endometrial curettage revealed moderately differentiated squamous cell carcinoma, signifying local extension of the cervical carcinoma into the endometrium (Fig. 3). Excision biopsy of a left cervical lymph node revealed metastatic deposits of moderately differentiated squamous cell carcinoma similar to that of the uterine cervix (Fig. 3). Ultrasound-guided fine needle aspiration cytology of intra-thyroid nodules revealed malignant squamous cells (Fig. 3). This was followed by a panendoscopy of the upper aerodigestive tract which yielded negative results.

A multidisciplinary meeting with the oncologists, surgeon, gynaecologists, pathologists and radiologists was conducted. Considering the histopathological and imaging findings with negative panendoscopy, a diagnosis of squamous cell carcinoma of the uterine cervix metastasising to the cervical lymph nodes and thyroid gland was made. A decision was made to commence palliative chemoradiotherapy. After 6 months, the patient developed generalised body oedema. She was transferred to a specialised cancer treatment centre for further palliative management, where she passed away a short while later, due to acute renal failure secondary to ureteric obstruction.

## Discussion

Metastatic tumours of the thyroid gland are a rare phenomenon [2]. Of them, primary gynaecological malignancies are even rarer, accounting for only 3% of metastatic thyroid cancers [3]. Metastasis to the thyroid may occur via lymphatic spread from the adjacent supraclavicular or cervical nodes or though haematogenous spread [4]. Clinical presentation of metastasis to the thyroid may vary. Although patients may present with a palpable thyroid nodule similar to the reported case, others are diagnosed incidentally during imaging techniques. In a study by Papi et al., about 70% of metastatic lesions of the thyroid gland presented with a palpable neck swelling, while about 30% of the cases were identified incidentally on imaging [5].

The most common sites for metastases arising from cervical cancer include the abdominal cavity, para-aortic lymph nodes and bone [2]. Cervical cancer metastasising to the thyroid gland is very rare and only a handful of cases have been reported [2]. These cases are summarised in Table 1 [2, 4, 6–9]. Sometimes, it may be clinically difficult to differentiate the origin of the tumour, whether it is primary or secondary. In doubtful cases, the origin of the malignant cells can be identified based on the cell morphology and immunocytochemical staining techniques. Immunocytochemical staining techniques involve the utilisation of anti-thyroglobulin and anti-calcitonin antibodies to stain the tumours. Negative staining with these antibodies would confidently rule out a thyroid origin [2].

**Table 1:** Summary of previous similar case reports

Author (year)	Clinical presentation	Biochemistry	Imaging	Histology	Treatment	Outcome
Martino (1977)	A 39-year-old female with thyroid enlargement for 2 months. Radical hysterectomy and lymphadenectomy for a stage I cervical SCC 2 years ago	Basic biochemistry: normal. Thyroid profile and uptake scan: normal Anti-Tg Ab: undetectable	CXR: sharply outlined nodule in right lung hilum	The thyroid biopsy specimen showed a poorly differentiated SCC	Thyroidectomy was abandoned due to a large mass with gross infiltration of the trachea and the left neurovascular structures. A biopsy specimen was obtained	Died in 4 months due to rapid enlargement of the tumour and metastases. Post-mortem examination showed wide-spread metastasis
Singh (2002)	A 38-year-old female with a rapidly growing neck mass. Radical hysterectomy and adjuvant chemoradiation for a stage IB neuroendocrine cervical carcinoma 1 year ago	Blood counts, basic biochemistry and thyroid profile were normal	MRI: large mass in the right side of the neck, which replaced the right thyroid lobe, and bilateral apical lung masses. CT: Multiple lesions in the liver	Biopsy of the thyroid and liver lesions revealed a poorly differentiated carcinoma. IHC panel was compatible with a neuroendocrine cervical carcinoma	Chemotherapy was initiated but developed significant adverse effects with the fourth cycle	Died 6 months after the diagnosis
Karapanagiotou (2006)	A 68-year-old female with cough, haemoptysis painless palpable thyroid swelling. History of Stage IIIB cervical SCC 4 years ago	Blood count and basic biochemistry: Normal ESR: 65	CT: large irregular mass (9 cm in diameter) in the neck, thyroid enlargement, enlarged mediastinal and para-aortic nodes, and multiple patchy lung infiltrates, no local recurrence Bone scan: negative	Bronchoscopy washing and brushing was positive for undifferentiated carcinoma Biopsy of the thyroid gland was performed, which showed non-keratinising SCC	6 cycles of systemic chemotherapy and local radiotherapy	Died 16 months after the diagnosis
Fuentes-Martinez (2015)	A 36 year-old woman with a thyroid nodule for one month. Radical chemotherapy for a hardly differentiated stage IA cervical carcinoma 1 year ago	NA	CT: an irregular nodule measuring 5 cm that occupied a large part of the right thyroid lobe	Fine needle aspiration showed hardly differentiated malignant cells	The patient received palliative treatment with radiotherapy and chemotherapy	Died 6 months after diagnosis
Celik (2016)	A 56-year-old female with thyroid lumps and dysphagia. Radical hysterectomy and adjuvant chemoradiation for a cervical SCC 6 months ago	Basic biochemistry, thyroid profile, Tg, anti-Tg Ab: normal	USS: multiple thyroid nodules with micro- and macro-calcifications PET/CT: multiple lung, spine, lymph node metastasis	Multiple islands of atypical mitotically active squamous cells in the thyroid	Palliative total thyroidectomy and central lymph node dissection	Died in 5 months due to rapid progressions of the disease
Varli (2018)	A 55-year-old female with a painless enlargement in the thyroid gland causing dyspnoea. She underwent radical surgery and chemoradiation for a stage IIA poorly differentiated cervical SCC	Basic biochemistry: normal. TSH was 0.02 IU/ml. Her fT3, fT4 and thyroid autoantibodies were normal	Ultrasonography: multinodular goitre. Pre-treatment PET/CT showed thyroid gland abnormalities with retrosternal elongation and metabolically inactive nodules, largest: 3 cm	Total thyroidectomy showed metastasis with squamous differentiation which was also identical to her treated cervical tumour	Total thyroidectomy	NA

*SCC* squamous cell carcinoma, *NA* not available, *Tg* thyroglobulin, *Ab* antibody, *MRI* magnetic resonance imaging, *CT* computed tomography, *PET* positron emission tomography, *IHC* immunohistochemistry

Similar to the previous reported patient, the pathological findings of the cervical lymph nodes and the thyroid were compatible with the primary squamous cell carcinoma of the uterine cervix [2]. Furthermore, the occurrence of squamous cell carcinoma of the thyroid is extremely rare and the presence of multiple similar deposits in the thyroid gland is also against the diagnosis of a primary squamous cell carcinoma of the thyroid. The negative panendoscopy ruled out a possible carcinoma arising from the upper aerodigestive tract. Although the mixed density mass in the left thyroid lobe may be a directly invading paratracheal metastatic lymph nodes, there were additional bilateral low-density intra-thyroid nodules (Fig. 2) and fine needle aspiration of these intra-thyroid nodules showed squamous cell carcinoma (Fig. 3A). Therefore, in our patient, a diagnosis of carcinoma of the uterine cervix metastasising to the thyroid gland was made after a multidisciplinary team discussion.

While metastases to the thyroid is considered to be indicative of very poor prognosis by some authors [3, 10], others believed that the prognosis would depend on the intrinsic characteristics and biology of the primary tumour [1]. The place of surgery for metastasis to the thyroid gland is controversial. The decision for surgery for thyroid metastases has to be decided on a patient-to-patient basis. Factors to consider include the degree of tumour metastases elsewhere, the overall condition of the patient, and the need to improve quality of life (such as respiratory and deglutition functions) [10]. However, our patient had extensive metastasis along the chain of lymph nodes in the abdomen, chest and neck in addition to the thyroid metastasis. Therefore, considering the above factors and the locally advanced primary tumour in the uterine cervix, a consensus was reached for palliative chemoradiotherapy.

In Sri Lanka, cervical cancer is the second most common cancer in females only second to breast cancer [11–13]. According to GLOBOCAN estimates, thyroid cancers are the third commonest cancer among Sri Lankan females; however, the incidence of metastatic cancers to the thyroid is unknown [13, 14]. Due to the relatively high prevalence of cervical cancer in Sri Lanka, nationwide screening programmes are freely available for the early detection [15]. However, these services are underutilised especially in the rural areas leading to late presentation of cervical cancer similar to the reported case. Therefore, more awareness among the public and primary care providers is necessary.

## Conclusion

Patients who present with a thyroid nodule along with multiple cervical lymph nodes should be thoroughly assessed for possible metastatic cancers. Cytological assessment provides a quick, and reliable pathway for the diagnosis, while histopathological evaluation of other suspected lesions is key to establish the final diagnosis. Metastasis to the thyroid gland indicates a poor prognosis in the background of carcinoma or the uterine cervix. More awareness among the public and primary care providers is necessary regarding freely available screening programmes for early detection of cervical cancer.

## Data Availability

Not applicable.
